# New Primary and Secondary Prevention Measures for Cutaneous Melanoma: A Systematic Review

**DOI:** 10.1002/cnr2.70698

**Published:** 2026-10-01

**Authors:** Maria Palau–del–Valle, Elena Bendala–Tufanisco, María Amparo López–Ruiz

**Affiliations:** ^1^ Graduate Student of Medicine Degree. Health Sciences Faculty Universidad Cardenal Herrera – CEU Valencia Spain; ^2^ Department of Biomedical Sciences Universidad Cardenal Herrera – CEU Valencia Spain

**Keywords:** AI‐based tools, chemoprevention, cutaneous melanoma, genetic association, in situ biomarkers, primary prevention, screening programs, secondary prevention

## Abstract

**Background:**

Cutaneous melanoma is the deadliest type of skin cancer, with increasing incidence and mortality rates. Therefore, primary and secondary prevention are of utmost importance.

**Claims:**

The objective is to evaluate new recent evidence on primary and secondary prevention strategies for cutaneous melanoma.

**Methods:**

Search of PubMed, Web of Science and EBSCOhost databases retrieved studies from February 2020 to January 2026, on prevention at both primary and secondary levels and that followed the PICO criteria selection process in the PRISMA method for a systematic review. Different assessment tools were used to evaluate methodological quality.

**Results:**

A total of 404 citations were analysed, of which 24 studies met the inclusion criteria: 3 addressed primary prevention and 21 secondary prevention. Primary prevention found a possible chemopreventive agent and the possibility of a genetic control of the mechanisms of nevi fluctuation in life associated with height. The secondary prevention studies evaluated population‐based screening strategies and emerging diagnostic tools, artificial intelligence (AI)‐assisted tools included, and found a new wearable microneedle biosensor for in situ tyrosinase activity detection as a new approach for early cutaneous melanoma detection. It is clearly a limited primary prevention publication that shows poor new evidence for conclusions.

**Conclusions:**

Two new primary prevention approaches were identified, but both require further research before clinical implementation: aspirin as a potential chemoprotective agent and a possible genetic association between height and age‐related nevus regression. Both require further study. Emerging diagnostic technologies, including AI‐assisted tools and wearable biosensors for in situ tyrosinase detection, demonstrated high sensitivity but variable specificity, requiring further validation before routine clinical use. Population‐based screening, patient education and risk‐stratified surveillance improved early melanoma detection, particularly among high‐risk populations. Most studies achieved moderate to high methodological quality, but diagnostic accuracy studies revealed some issues showing that require relevant improvements in diagnostic accuracy and standardization. The use of biomarkers is a promising new tool for early diagnosis.

## 1 Introduction

Cutaneous melanoma (CM) is a malignant tumor arising from melanocytes [1], pigment‐producing cells located in the basal layer of the epidermis. Although CM accounts only a small fraction of all skin cancers (4%), it accounts for the vast majority of skin cancer related deaths (80%), due to its aggressive behavior and high metastatic potential [2].

The pathway from melanocytes to melanoma, termed melanomagenesis, is a multifactorial process involving several factors, ranging from environmental exposures to genetic alterations [2]. Ultraviolet (UV) radiation is the most established external risk factor [3, 4, 5], particularly intense intermittent exposure [6]. UV–Induced DNA damage disrupts tumor suppressor mechanisms and leads to oncogenic mutations [7, 8]. Indoor tanning is also consistently associated with increased melanoma risk, particularly for early‐onset disease [9]. Internal risk factors include fair skin phototypes (I–III) [10], genetic mutations such as the BRAF^V600E^ and NRAS^Q61K/R^ oncogenes, immunosuppressive states, chronic inflammation, and advancing age [1, 11]. Emerging Mendelian randomization evidence also implicates the gut microbiome as a potential causal contributor to melanoma risk [11].

Regarding classification systems, at the current time the 2018 WHO classification identifies nine melanoma subtypes based on genetic and histological features, with seven from these considered cutaneous in origin [12]. The traditional CM classification (according to their growth pattern) classifies CM into primary and metastatic. Primary CM melanoma is further divided into melanoma in situ and invasive. Lastly, invasive CM is classified according to its clinical and histopathological characteristics into 4 major subtypes: superficial spreading melanoma, nodular melanoma, lentigo maligna melanoma and acral melanoma, being the former the most common with 41% of cases [13].

Clinically, CM can develop de novo or from existing pigmented lesions, the ABCDE criteria remaining central to clinical suspicion, and the Breslow thickness serving as a key prognostic marker [14].

Epidemiological trends reflect a growing burden worldwide. According to the latest available data from the “Global Cancer Observatory (Globocan)”, over 330 000 new cases of CM were reported in 2020, with nearly 59 000 deaths. These values translate into a 17.7% mortality rate [15]. The annual incidence rates increase each year from 3% to 7%, resulting in a doubling every 10–20 years [16]. Incidence is highest in fair–skinned populations residing in regions with high UV exposure such as Australia, Europe, and North America. While mortality rates appear to plateau in some high–income countries, a new study from the “International Agency for research on Cancer (IARC)” predicts an increase of more than 50% in the number of new cases of melanoma per year from 2020 to 2040 [17].

Preventive measures are typically stratified across four levels: primordial (policy–level changes in order to prevent the exposure of society to risk environmental or social factors since childhood), primary (individual behavior modification on susceptible populations to be exposed to a risk factor able to produce illness), secondary (early detection of the illness in order to reduce the damage; screening procedures for early detection fall into this category), and tertiary (to lessen the impact of an existing disease on the patient's quality of life and longevity) [18, 19]. The tertiary prevention in the case of cancer entails a high economic cost and a social and personal burden that is always excessive. Hence the need to strengthen measures taken before the disease develops. In CM there are some traditional established prevention measures, such as the use of sun protection, but more recent advances and approaches remain underexplored in recent reviews [20].

As an urgent need for effective prevention strategies is highlighted [21], this systematic review aims to evaluate current evidence (2020–2025) on the effectiveness of primary and secondary prevention strategies for cutaneous melanoma in the general population with the newest advance in the field. By integrating emerging data and assessing the quality and outcomes of recent interventions, the review seeks to inform clinicians, policymakers, and public health stakeholders in optimizing melanoma control efforts.

## 2 Materials and Methods

### 2.1 Review Design

This systematic review was conducted in accordance with the PRISMA 2020 guidelines [22] and the Synthesis Without Meta–analysis (SWiM) reporting framework [23] to ensure methodological transparency and rigor. The study protocol and detailed search strategy were registered in the PROSPERO database (CRD420251109488).

### 2.2 Eligibility Criteria

The study was structured around a predefined PICO question (In the general population, what are the new primary prevention measures proposed since February 2020 to January 2026 years for the reduction on the incidence of cutaneous melanoma skin cancer? And which secondary prevention measures have demonstrated to be effective in CM screening?) as is explained in Supplementary Table 1. Only original research articles with complete methodology and results were included; commentaries, letters to the editor, narrative opinions, and incomplete reports, all the grey literature, were excluded. All inclusion and exclusion criteria are provided in Supplementary Tables 2 and 3.

### 2.3 Search Strategy

A systematic search was conducted independently by two authors on February 3rd 2025 and repeated on June 6th 2026, on the Web of Science, PubMed and EbscoHost databases, covering in the last search studies from February 2020 until January 2026. Search terms included combinations of “cutaneous melanoma”, “primary prevention”, “secondary prevention” and “screening”. Complete search strategies for each database are shown in Supplementary Tables [Link], [Link].

The results were retrieved and imported into the Refworks software, where after removing the duplicates, two independent researchers performed the selection process. All titles and abstracts were screened, and full texts of potentially relevant studies were assessed against inclusion criteria. Discrepancies were resolved by consensus or arbitration by a third reviewer, who supervised all the searches. Studies accounting for previous reviews or assessing tertiary prevention strategies were excluded.

### 2.4 Data Extraction and Analysis

Data were extracted independently by two reviewers using a standardized data collection form. The extracted variables for each study included: study characteristics (first author, year of publication, study design, country), participant details, intervention type, objectives, comparators, results (incidence, tumor thickness at diagnosis, stage at diagnosis, detection rates), and conclusions. Given the methodological and clinical heterogeneity across studies, the obtained data were used to populate summary tables to inform a narrative synthesis.

### 2.5 Assessment of Study Quality and Certainty of Evidence

The quality of each included study was independently assessed by two reviewers using a designed‐specific appraisal tool: the Cochrane Risk of Bias 2.0 tool (Rob–2) [24] for Randomized Controlled Trials (RCTs), the Newcastle–Ottawa Scale (NOS) [25] for observational studies, the QUADAS–2 scale [26] for diagnostic accuracy studies, the JBI Critical Appraisal Checklist for Analytical Cross‐Sectional Studies [27] and the NHI Quality Assessment Tool for Before‐After (Pre‐Post) Studies with no control group [28] for the single quality improvement study lacking a control group.

The certainty of the body of evidence for each outcome was evaluated using the GRADE (Grading of Recommendations Assessment, Development and Evaluation) approach [29]. Evidence was classified as high, moderate, low, or very low, based on study limitations, inconsistency, indirectness, imprecision, and publication bias. Results are presented in summary of findings tables.

## 3 Results

Database searches retrieved 404 records, of which 53 were duplicates. After performing title and abstract screening, 92 underwent full text assessment, 24 studies met the eligibility criteria: 3 evaluated primary prevention strategies and 21 evaluated secondary prevention measures. The studies were published between February 2020 and January 2026 and originated from diverse geographic regions including North America, Europe and Asia. Study designs included 1 RCTs [30], and 23 observational studies that comprise retrospective, prospective, longitudinal and cross‐sectional designs, including nine diagnostic accuracy studies [31, 32, 33, 34, 35, 36, 37, 38, 39], 13 conventional observational studies [40, 41, 42, 43, 44, 45, 46, 47, 48, 49, 50, 51, 52] and one before–after quality improvement study [53]. The following PRISMA flowchart (Figure 1) shows the multi‐stage study acquisition process, as well as the reasons for exclusion.

**FIGURE 1:**
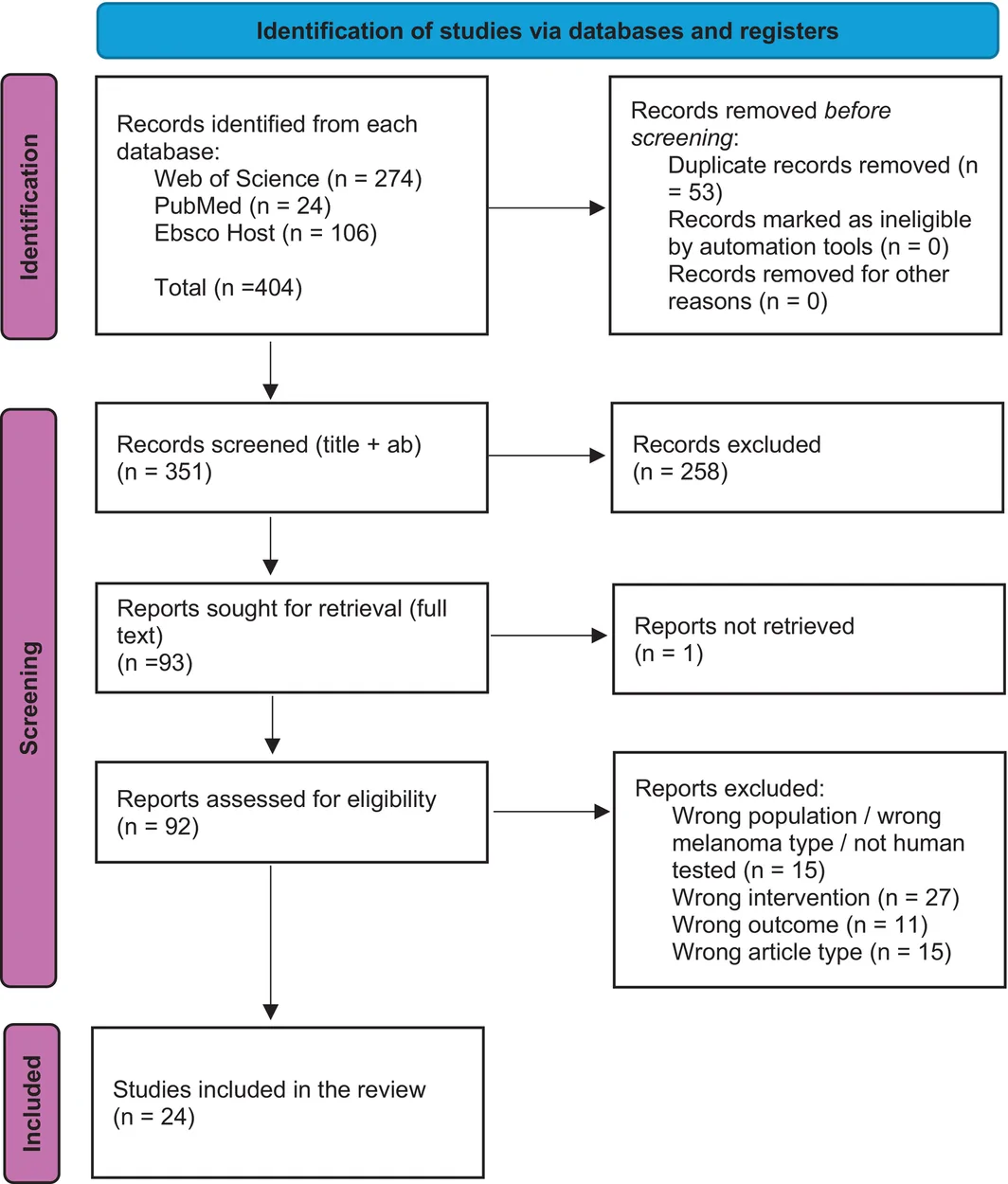
PRISMA Flow Diagram (2020) illustrating the study selection process for this systematic review.

A comprehensive summary of the included studies regarding primary prevention can be seen in Table 1, of the included studies regarding secondary prevention in Table 2 and of the interventions and results of all included studies in Table 3.

**TABLE 1:** Summary of included studies regarding primary prevention.

Title and authors	Year and country	Study type	Participants	Objectives	Outcome
Association between hypertension and cutaneous melanoma, and the effect of aspirin: extended follow‐up of a large randomized controlled trial. M. K. Yan et al. [40]	May 2022 Australia	Retrospective observational study of a previous Randomized Controlled Trial	19 114	Assess the effect of aspirin on melanoma development across hypertensive patients.	Reduced melanoma incidence among hypertensive individuals taking aspirin.
Effect of aspirin on melanoma incidence in older persons: extended follow‐up of a large randomized double‐blind placebo‐controlled trial. M. K. Yan et al. [41]	June 2022 Australia	Retrospective observational study of a previous Randomized Controlled Trial	19 114	See if aspirin associates with a lower risk of CM in older individuals.	Support (but not strong) that aspirin acts as a chemopreventive factor for cutaneous melanoma.
An Anthropometric Study of Total Body Nevus Counts, Height, Sex, and Age Differences C. Dessinioti et al. [42]	February 2025 Greece	Observational Cross‐sectional study	813	Assess nevus counts	Older age associated with fewer nevi and decrement of number of nevi as higher stature with age and possibly lower in women

**TABLE 2:** Summary of included studies regarding secondary prevention.

Title and authors	Year and country	Study type	Participants	Objectives	Outcome
Automatized self‐supervised learning for skin lesion screening. Useini et al. [31]	June 2024 Switzerland	Cross‐sectional observational study (diagnostic accuracy)	44 images and 17 clinical validation participants	Develop a total body screening tool that achieves expert‐level accuracy in identifying and classifying lesions through wide field images.	The AI tool achieved a 95% sensitivity identifying lesions, and younger doctors with less experience also improved their sensitivity.
Clinical Outcomes of Free, Public Skin Cancer Screening Events, Rhode Island, 2015–2019. Lee et al. [43]	August 2021 USA	Retrospective observational study	2354	Promote skin cancer awareness in the general population. Evaluate the effectiveness of free screening in the early detection of melanoma and other skin cancers.	Free screening in beaches can increase early detection of skin cancer, in those populations with high sun exposure. It is recommended to continue such programs and to improve the follow‐up rate to confirm diagnoses.
Diagnostic performance of augmented intelligence with 2D and 3D total body photography and convolutional neural networks in a high‐risk population for melanoma under real‐world conditions: A new era of skin cancer screening? Cerminara et al. [32]	June 2023 Switzerland	Prospective study (diagnostic accuracy)	143	Assess the efficacy of three‐dimensional (3D) and two‐dimensional (2D) Convolutional neural networks (CNNs) and dermatologists in the early detection of melanoma in a real‐world setting.	The 3D‐CNN device outperformed the 2D‐CNN and achieved comparable sensitivity with dermatologists. However, the low specificity of CNNs currently limits their use in clinical practice.
Five‐Year Outcomes of a Melanoma Screening Initiative in a Large Health Care System. Matsumoto et al. [44]	April 2022 USA	Retrospective observational study	595 799	Evaluate if the screening for CM in a primary care setting is associated with a reduction in the diagnosis of thicker lesions	There was a higher incidence of thin melanomas among screened patients, but also a lower incidence regarding thick melanomas.
Implementation of artificial intelligence algorithms for melanoma screening in a primary care setting. Giavina‐Bianchi et al. [33]	September 2021 Brazil	Retrospective observational study (diagnostic accuracy)	3083 clinical images	Development of an AI system for cutaneous melanoma screening in a primary care setting	2 AI models were created, and both achieved an area under the curve of > 0.9, indicating a high discriminative capacity.
Lesion‐directed screening to optimize skin cancer detection in dermatology practice: an observational study. Mylle et al. [45]	2021 Belgium	Observational study	297	Study if a lesion‐directed early‐access consultation after triage by phone can optimize skin cancer detection in dermatology practice.	The results of the study showed high overall skin cancer and melanoma detection rates. Besides surveillance programs in high‐risk patients, lesion‐directed screening could optimize skin cancer detection in the general population.
Remote skin self‐examination training of melanoma survivors and their skin check partners: A randomized trial and comparison with in‐person training. Robinson et al. [30]	June 2020 USA	Randomized controlled trial (RCT)	341 melanoma survivors and their partners	To compare the effectiveness of remote versus face‐to‐face training on knowledge and frequency of self‐examinations. To assess whether remote training improves detection of suspicious melanoma lesions.	Remote training in skin self‐examination is a viable and effective alternative to face‐to‐face training. Its use is justified as an adjunct to melanoma surveillance to improve early detection and reduce the burden on health services.
*Papel de la Farmacia Comunitaria en la Prevención Secundaria del Cáncer de Piel*. A. González et al. [46]	January 2025 Spain	Transversal observational study (diagnostic accuracy)	41	Early detection of melanoma and non‐melanoma skin cancer in patients visiting the pharmacy. Facilitate referral to dermatologists through the community pharmacy. Optimize the use of healthcare resources, avoiding unnecessary consultations.	More than 85% of the preliminary diagnoses were accurate from the submitted images alone.
Screening for Melanoma and Other Skin Cancer Shows a Higher Early Melanoma Incidence: Social Educational Program “Life Fear‐Free” L. Demidov et al. [47]	March 2021 Russia	Cross‐sectional observational study	3143	To analyse the impact of the program on early CM and non‐melanoma skin cancer (NMSC) detection.	The program increased detection of melanoma at earlier stages but demonstrated low melanoma awareness in the population. It is recommended to expand the campaign to include higher risk populations and to improve adherence to the second phase of screening.
The impact of longitudinal surveillance on tumor thickness for melanoma‐prone families with and without pathogenic germline variants of *CDKN2A* and *CDK4* Sargen et al. [48]	October 2021 USA	Longitudinal observational study	293	To assess whether medical surveillance and self‐examination reduce tumor thickness at diagnosis. To compare outcomes in families with and without CDKN2A and CDK4 mutations.	Long‐term follow‐up with clinical examination and self‐examination education reduced melanoma thickness at diagnosis. 83% of melanomas in the Prospective group were classified as T1 (lesser thickness and better prognosis), compared to 40% in the Pre‐study group (*p* < 0.001).
Three‐Dimensional Total Body Photography (TBP), Digital Dermoscopy (DD), and in vivo Reflectance Confocal Microscopy (RCM) for Follow‐Up Assessments of High‐Risk Patients for Melanoma: A Prospective, Controlled Study. Hobelsberger et al. [49]	October 2024 Germany	Prospective observational study	600	To determine whether the combination of 3D‐TBP, DD and RCM improves melanoma detection compared to dermoscopy alone. To assess whether these technologies reduce the need for unnecessary biopsies. To measure the impact on the detection of second malignancies in patients with a history of melanoma.	Follow‐up with 3D‐TBP, DD and RCM significantly improve the early detection of melanoma compared to dermoscopy alone. In addition, RCM reduces the number of biopsies required.
Using Artificial Intelligence as a Melanoma Screening Tool in Self‐Referred Patients. Crawford et al. [34]	December 2023 Canada	Prospective observational study (diagnostic accuracy)	318	To assess the accuracy of AI in detecting melanoma in self‐referred patients. To compare the sensitivity and specificity of AI against expert dermatologists. To determine the feasibility of including AI in melanoma screening programs to improve access to early diagnosis.	It detected 11 of 17 malignant lesions. It did not identify 6 melanomas in situ, which were also difficult for dermatologists and dermatopathologists to diagnose. AI sensitivity: 64.7% AI specificity: 75.8%, comparable to specialists.
Validation of a Market‐Approved Artificial Intelligence Mobile Health App for Skin Cancer Screening: A Prospective Multicenter Diagnostic Accuracy Study. T. Sangers et al. [35]	February 2022 Netherlands	Prospective observational study (diagnostic accuracy)	372	To evaluate the sensitivity and specificity of the application for the detection of premalignant and malignant lesions. Determine whether AI can be a useful tool to empower patients to self‐assess skin lesions before consulting a specialist.	The AI‐based application showed moderate‐high diagnostic accuracy, with comparable sensitivity to that of general practitioners. However, its specificity is still low, which could lead to unnecessary consultations. Further validation in suspicious pigmented lesions and in different skin types would be recommended before considering mass implementation in the general population.
Clinical ABCDE rule for early melanoma detection Duarte et al. [50]	December 2021, Portugal	Retrospective observational study	144 melanomas	To assess the usefulness of the ABCDE rule in early melanoma detection	Helpful for clinical suspicion but may miss some very early melanomas
Combining hyperspectral imaging techniques with deep learning to aid in early pathological diagnosis of melanoma C. Tian et al. [36]	September 2023, China	Retrospective experimental study using AI‐assisted hyperspectral imaging. Diagnostic accuracy	100 patients (50 melanomas, 50 pigmented nevi)	To evaluate the ability of hyperspectral imaging and deep learning to differentiate melanoma from pigmented nevi	Diagnostic accuracy of 89% (1D data) and 98% (2D data)
Factors associated with suspected nonmelanoma skin cancers, dysplastic nevus, and cutaneous melanoma among first‐time SpotMe screening participants Beaulieu et al. [51]	January 2023, United States	Cross‐sectional observational study	118 085 participants	To identify demographic and clinical factors associated with melanoma and other skin cancers	Factors associated with suspected nonmelanoma skin cancers, dysplastic nevus, and cutaneous melanoma among first‐time SpotMe screening participants (Beaulieu et al.)
Self‐Assessment Questionnaire on Patient–Physician Concordance on Nevus Self‐Count and models development to predict High risk phenotype. Mannino et al. [52]	September 2022, Italy/Spain	Cross‐sectional	Adults	Assess self‐count of nevi	Back nevi predicted high‐risk phenotype
Evaluation of human melanoma and normal formalin paraffin‐fixed samples using Raman and LIBS fused data. Khan, MN et al. [37]	July 2022, China/USA	Diagnostic accuracy study	Tissue samples	Improve detection	High diagnostic accuracy
In Situ Tyrosinase Monitoring by Wearable Microneedle Patch toward Clinical Melanoma Screening. Huang X et al. [38]	October 2023, China	Experimental	Animal/human samples	Develop detection device	Accurate melanoma detection
Improving Skin Cancer Knowledge and Screening Among Older Chinese Americans. Wang Y et al. [53]	November–December 2024, USA	Cross‐sectional	77 participants	Increase screening Quality improvement	Knowledge improved + will to receive TBE
A Thermal‐Imaging System and Machine‐Learning Classification Algorithm for Skin Cancer Screening. Mainardi V. et al. [39]	August 2025, Italy	Prospective diagnostic accuracy study	58 patients with benign and malignant skin lesions	To develop and validate a non‐invasive thermal‐imaging system combined with a machine‐learning algorithm for skin cancer screening and early melanoma dtection.	The system achieved 89.7% accuracy in distinguishing benign from malignant lesions and 95.5% accuracy in differentiating melanoma from nevi, demonstrating its potential as a non‐invasive support tool for early skin cancer screening.

**TABLE 3:** Summary of the intervention and results of all included studies.

Study	Intervention	Results	Determination of results
M. K. Yan et al. [40] (Association between…)	Randomization to daily 100 mg of aspirin or placebo.	Hypertension with aspirin associated with less melanoma risk (Hazard ratio (HR) = 0.73, 95% confidence interval (CI) 0.55–0.97) Higher risk in patients with uncontrolled hypertension	Histopathology. Cox model.
M. K. Yan et al. [41] (Effect of aspirin…)	Randomization to daily 100 mg of aspirin or placebo.	No statistically significant differences between the 2 groups (HR = 1.04, 95% CI: 0.77–1.40)	Histopathology. Cox model, Kaplan–Meier curves and sensitivity analyses.
C. Dessinioti et al. [42] (An Anthropometric stu.)	Total body nevus counts manually performed by dermatologists and data collection.	People older than 51 years had 73% lower likelihood to have 30–60 nevi, but if they were 10 cm or more taller than the average, lower likelihood of having 30 or more nevi (OR: 0.57, 95% CI: 0.33–0.998, *p* = 0.049). The difference with age and height was statistically significant in women *p* < 0,001	Visual inspection and count Phenotype assignment based on skin color and survey responses regarding sensitivity to sun exposure
Useini et al. [31] (Automatized self‐supervised),	AI based decision tool with a skin lesion detector (YOLOR‐P6) and an Ugly Duckling detector (DINO)	95% sensitivity for the top 10 suspicious lesions identified by experts. 100% accordance between the AI and the experts.	Histopathology. Statistical analysis using recall, precision, Average Precision (AP) and Average Recall (AR) to measure performance.
Lee et al. [43] (Clinical Outcomes of Free, Public.)	27 free skin cancer screening events held on Rhode Island beaches during peak UV hours in July or August.	25% of screened patients referred for dermatological follow‐up. Number needed to biopsy (NNB) = 3, Number needed to screen (NNS) = 18.3	Histopathology. Data analysis using Microsoft Excel and comparing with previous studies.
Cerminara et al. [32] (Diagnostic performance of augmented…)	TBP using 3D AI (Vectra WB360) and 2D AI (FotoFinder ATBM) with posterior application of 2D and 3D CNN	3D AI: 90% sensitivity and 64.6% specificity 2D AI: 70% sensitivity and 40% specificity Accuracy of dermatologist was superior to 2D AI, but comparable to 3D AI. The combination of dermatologist +3D AI improved diagnostic accuracy.	Histopathology. Total body photography devices (FotoFinder ATBM and Vectra WB360) ROC analysis, Pearson correlation test and logistic regression model.
Matsumoto et al. [44] (Five‐Year Outcomes of…)	Full‐body screen examinations performed by primary care clinicians on eligible patients.	Thin invasive melanoma (≤ 1 mm) was more frequently diagnosed in the screened group (incidence: 24.5 vs. 16.1, *P* < 0.001) For thicker melanomas (> 2 mm), incidence was lower among screened patients, but these differences did not reach statistical significance (*p* > 0.05)	Histopathology. Cox model and Kaplan–Meier curves.
Giavina‐Bianchi et al. [33] (Implementation of artificial…)	Implementation of 2 AI computer‐aided diagnosys (CAD) systems in primary care. Clinical and dermoscopic models.	Dermoscopic AI: 91% sensitivity and 89% specificity Clinical AI: 89% sensitivity and 83% specificity	Histopathology. ROC analysis, *t*‐test, Chi‐square and logistic regression. Cross‐validation using external data sets (ISIC2019 and PH2).
Mylle et al. [45] (Lesion‐directed screening…)	Triage via a form. Eligible patients offered an early‐access consultation with TBP	Melanoma detection rate: 4.1% overall (9% in patients advised/referred by a doctor, 7.7% in patients with no referral and a negative personal history). TBE is significant only when the index lesion is suspicious, in cases with benign index lesions. Anxiety: in patients with benign lesions, the mean VAS score dropped by 3.6 points versus a 1.2‐point drop in those with suspicious lesions.	Histopathology. Dermoscopy and TBE Chi‐square, *t*‐test and logistic regression.
Robinson et al. [30] (Remote skin self‐examination…)	Phase 1: Training in self‐exploration (SSE) through a workbook, diagrams and scorecards Phase 2: quarterly follow‐up in the face‐to‐face phase and through surveys in the remote phase.	Intervention group performed more SSE than controls in both phases (*p* < 0.05) Frequency of SSE ranged from 2.38 to 5.97 times in 9 months. The intervention group detected more suspicious lesions and melanomas than the control group. Remote training was equally effective as in‐person training.	Histopathology and direct clinical revision. Analysis of Variance (ANOVA)
González et al. [46] (Papel de la Farmacia…)	Questionnaire and 2 photographs sent through Bidafarma platform (Telederma)	54% had benign lesions and 46% had lesions suspicious for cancer. Sixty‐three percent of suspicious lesions were in patients > 60 years old. More than 85% of the preliminary diagnoses were accurate based on imaging analysis.	Preliminary analysis with teledermatology. Histopathology after dermatology consultation.
Demidov et al. [47] (Screening for Melanoma…)	Educational campaign and skin examination; suspicious lesions followed by excisional biopsy.	3 cases of cutaneous melanoma detected (all with Breslow thickness of 1 mm). Higher incidence rate of cutaneous melanoma (95.45 per 100 000) compared to the national average (7.76 per 100 000)	Histopathology. Comparison of detection rates with the general population.
Sargen et al. [48] (The impact of longitudinal…)	Routine skin cancer screening and education about skin self‐exams	Mean thickness for prospective melanomas: 0.6 mm Mean thickness for pre‐study melanomas: 1.1 mm Higher percentage of early‐stage melanomas (T1 stage): 83% of prospective cases were T1 stage versus 40% of pre‐study cases	Histopathology. Logistic and linear regression and mixed‐effects model. Comparison with SEER registry data using Peters‐Belson model.
Hobelsberger et al. [49] (Three‐Dimensional Total…)	Intervention group: 3D‐TBP at every visit. DD and/or RCM performed when lesions were suspicious or changed in appearance	3D‐TBP, dermoscopy and RCM detected more melanomas (22 vs. 1; *p* = 0.022). Superior sensitivity of RCM (NNT = 2.83) More nevi excised in the intervention group (186 vs. 10; *p* < 0.001), with more dysplastic nevi (137 vs. 2; *p* < 0.001).	Histopathology. Dermoscopy, TBP and RCM. Chi‐square, Fisher's and Mann–Whitney tests
Crawford et al. [34] (Using Artificial Intelligence…)	Assessment of skin lesions by AI (FotoFinder Moleanalyzer Pro) in self‐referred patients.	10 melanomas were identified out of 381 suspicious lesions. AI showed comparable accuracy to dermatologists (AUC = 0.73 vs. 0.70), detecting 11 of the 17 skin cancer cases, with a biopsy rate of 2.25 for melanoma. AI sensitivity: 64.7%. AI specificity: 75.8%	Histopathology. Double‐blind evaluation of results by dermatologists. ROC curves, chi‐square test and Kappa coefficient.
Sangers et al. [35] (Validation of a Market‐…)	Evaluation of skin lesions through the SkinVision (CNN) app. Posterior derivation to dermatology depending on the assessment.	Sensitivity = 86.9% Specificity = 70.4% Higher sensitivity on iOS devices (91.0%) versus Android (83.0%; *p* = 0.02). Higher sensitivity in skin folds (92.9%) than in smooth skin (84.2%; *p* = 0.01). Diagnostic accuracy was comparable to that of dermatologists.	Histopathology. SkinVision CNN and dermatologists cross‐checking. ROC curves and Chi‐square test.
Duarte et al. [50] (Clinical ABCDE rule…)	Recovery of data ABCDE from 144 diagnosed malignant cutaneous melanoma in the clinical histories for 11 years	The in situ melanoma had less ABCDE signs, but an increased number of ABCDE was not reducing the chance of a MM being diagnosed in situ (OR = 0.9; 95% CI = 0.7–1.1; *p* = 0.377). More ABCDE signs, higher the probability of diagnoses by non‐dermatologist < 0.001**	Histopathology and Dermoscopy.
C. Tian et al. [36] (Combining hyperspectral…)	Comparative study of images from 50 patients already diagnosed of cutaneous melanoma and 50 voluntary subjects with pigmented nevus.	Accuracy of pigmented tumors and pigmented nevi was 89% for one‐dimensional sample data and 98% for two‐dimensional sample data.	Microscopic hyperspectral imaging system (GaiaMicro‐G‐V10E‐AZ4, Dualix Spectral Imaging) analyzed by a computer integrating transfer learning with deep convolutional networks to classify melanomas and pigmented nevi in hyperspectral pathology images
Beaulieu et al. [51] (Factors associated with…)	Screening of different skin cancers with a A total of 1297 suspected CMs (1.1% of eligible) (screenees) were detected.	A positive association between age and suspected CM was observed (aOR 1.01, 95% CI 1.01–1.02). People with >[30 h of weekly sun exposure were with higher risk, men and people never attending to a dermatologist].	SpotMe skin cancer screening program No histopathological confirmation.
Mannino et al. [52] (Self‐Assessment Questionnaire…)	Evaluation of the perception of lesions of patients by self‐assessment questionnaire followed by total body skin examination	Implementation of skin self‐assessment questionnaires could be useful for melanoma secondary prevention. The single best predictor for cutaneous melanoma risk was the number of nevi on the back.	Self‐assessment questionnaire Total body skin examination by a dermatologist.
Khan, MN et al. [37] (Evaluation of human melanoma…)	Five melanoma cancer samples and five normal tissue samples from two patients were analysed with a hybrid spectroscopic system.	The data obtained were compared with different types of LIBS and Raman laser isolated or combined, showing that the combination of Raman spectroscopy and LIBS significantly enhances the discrimination and classification accuracy of melanoma and normal FFPE tissues using The ELM, an algorithm configured for feeding forward a single layer neural network. The classification accuracy was 99,33%	Biopsies from two already diagnosed patients were formalin‐fixed paraffin‐embedded on the glass slides and study with two sources and spectroscopic apparatus LIBS and Raman combined
Huang X et al. [38] (In Situ Tyrosinase…)	Development of a cutaneous sensor for measurement of tyrosinase activity in pigmented areas of the skin that could be maintain several days	The selection of Thyrosinase as a sensing melanoma‐related biomarker is innovative and measuring through the skin with this sophisticated system exhibited a good linear relationship of TYR with an ultralow detection of limit of 0.01 U/mL for SERS mode and 0.03 U/mL	in situ sensing with use of a microneedle array patch with 100 of tiny tips to reach deep skin areas and detect tyrosinase activity because the system comprises a Au@Ag nanosphere inside that determine TYR levels in human skin samples through a SERS/colorimetric dual‐mode method
Wang Y et al. [53] (Improving Skin Cancer…)	Educational program about skin cancer in a community of elderly Chinese Americans	The participants showed a significant improvement in their knowledge about skin cancer (*p* < 0.001), and they were less reluctant to let the nurse certified in dermatology to perform a total body skin examination	Culturally tailored skin cancer education program for Chinese Americans aged 65 and more years including sun protection behaviors and the ABCDE rules for self‐examination.
Mainardi V. et al. [39] (A Thermal‐Imaging System…)	In patients already diagnosed and selected for biopsy a screening with the association of thermal‐imaging system, and a software for analysis and classification of images was performed.	Accuracy of 89.7% in distinguishing between malignant and benign lesions in a case study of 16 cutaneous melanoma and 5 junctional melanocytic nevi classifying them with an accuracy of 95.5%.	Thermal‐imaging system, and the anonymized data were analysed through the thermal analysis algorithm, and classified using the implemented ML classification algorithm

### 3.1 Primary Prevention

Three studies investigated primary prevention: two of them were conducted as observational studies over the same previous RCT and mentioned aspirin as a chemopreventive agent. One large population‐based cohort study reported that long‐term aspirin use (≥ 5 years) was associated with a 14% reduction in cutaneous melanoma incidence compared with nonusers (adjusted hazard ratio [HR] 0.86; 95% CI 0.74–0.99), with the strongest effect observed among individuals with uncontrolled hypertension [40]. The second observational study, focused on aspirin's effect in older patients, found no statistically significant reduction in melanoma incidence with aspirin use [41]. No significant increase in adverse events was observed in either study.

The third study confirmed the previously described association between greater height and a more pronounced age‐related decline in total nevus count, particularly in women, which is interpreted as involving some genetic mechanisms in melanoma susceptibility, although no direct protective effect against cutaneous melanoma was demonstrated [42].

No articles about sun protection or the differences among the six Fitzpatrick skin phenotypes requirements were published in the studied period, showing no appearance of new sunscreen products or newly found environmental risk factors for melanoma development. This makes difficult the obtention of conclusions regarding primary prevention, but still we considered relevant the inclusion of these three articles considering their potential relevance in the future.

### 3.2 Secondary Prevention

Eight studies evaluated **AI algorithms** for CM detection in dermoscopy, total body photography, thermal‐imaging system [39] or histopathological analyses of biopsies of already diagnosed patients [37]. Convolutional neural networks (CNNs) and hybrid models consistently achieved high specificity (70,4% to 100%), often equal to that of expert dermatologists [32, 33, 34, 35]. This contrasts with their sensitivity levels, which varied considerably: from 58,35 in 2D TBP CNN models [32] to 89% in dermoscopic models [31, 33]. The diagnostic accuracy was between 70,5% and 99,33%.

The tools that were embedded into clinical settings with professional oversight, outperformed the standalone or consumer facing tools [34, 35]. Nonetheless, it is worth mentioning that several of these models were limited by “difficult” skin areas, such as those with tattoos, body folds, or regions with image quality challenges [31, 34, 35, 49], and that they only or predominantly included patients with higher risk phototypes (Fitzpatrick I‐II) [31, 32, 33, 35, 51, 52]. These constitute limitations on the applicability of the results, therefore, while promising, these tools require further refinement, particularly for improving their accuracy and validating performance across all skin phototypes in order to avoid underrepresentation precisely of the more vulnerable population.

To clarify the benefits of different IA procedures they were divided into three main categories: Classical Machine‐learning (ML), in which it requires manual features extraction from samples selected by dermatologists; Deep learning (DL), that has a “neuronal network” able to select relevant features from the samples in an automatic learning process; and Self‐supervised learning (SSL), the newest and most sophisticated procedure in which the system learns without labeled data produced by dermatologist. Table 4 shows the specific developed procedures for each technique.

**TABLE 4:** Type of artificial Intelligence used for clinical diagnosis and characteristics.

Type of artificial intelligence	Clasical machine learning	Deep learning	Self‐supervised learning
**Algorithms included**	Support Vector Machines (SVM widely used to detect differences between healthy and pathological tissues) Extreme Learning Machines (ELM algorithm configured for feeding forward a single layer neural network), Partial Least Squares Discriminant Analysis (PLS‐DA statistical technique to clarify massive complexity of data set) Random forest K‐Nearest‐Neighbors (K‐NN, that compares chemical unknown structures with known ones) Logistic regression, mainly used for classification	Convolutional Neural Networks (CNN) Residual Networks (ResNet), uses 50 CNNs EfficientNet, an improvement of CNNs Dense Convolutional Networks (DenseNet), a new way of arbitrary multiple layers of the image interconnections	Simple Framework for Contrastive Learning of Representations (SimCLR) Bootstrap Your Own Latent with two neuronal networks that learn from each other. (BYOL) Momentm Contrast (MoCo) DINO that uses Vision Transformers, improved in each new version.
**Requierement of labeled data**	Almost all the data should be labeled	Large amount of data labeled	No. The program learns from unlabelled data
**Feature extraction**	Manual, defined by trained dermatologist or other experts	Automatic	Automatic
**Level of human intervention**	High	Moderate	Low
**Data requierement and computational cost**	Low to moderate	High	Very high
**Typical applications in melanoma diagnosis**	Thermographic data, spectroscopic data, clinical variables	Dermoscopy, clinical imaging, histopathology, hyperspectral imaging	Automated screening, suspicious lesion detection, large‐scale image analysis
**Key advantage**	Performs well with limited data	High diagnostic accuracy	Reduces the need for expert image annotation
**Main disadvantage**	Depends on manually selected variables	Requires many labeled images High consumption of energy	Technical complexity and limited clinical validation High consumption of energy and water.

Table 5 groups the articles based on the type of artificial intelligence used to make easier to compare the data.

**TABLE 5:** Summary of articles using AI classified by type.

Title and authors	Secondary prevenion strategy	AI type	Specific technique	Applications	Outcome
Automatized self‐supervised learning for skin lesion screening. Useini et al. [31]	AI assisted screening of the real‐world data of total body screening (RWD TBS) obtained in revision of patients with more than 100 nevi with a final evaluation by experts	Self‐Supervised Learning comparing DINO (that prove superior) and MoCov2 associated to 3 ResNet	Object detection combined with self‐supervised learning	Automatic identification of suspicious lesions (“ugly duckling” lesions (UDs)) in RWD TBS images of only 11 patients comparing results of expert dermatologist and students with and without AI assistance.	In analysis of the patients by dermatologist of around 4 UDs lesions, the sensitivity was 80%–84%, and for students picking 7–8 UDs, 63%–68%. With AI assistance predictions achieve on average a sensitivity value of 95% and reach even 100% on average when presented to the expert and 81%–82% among the students. The worst results were found in hairy areas.
Diagnostic performance of augmented intelligence with 2D and 3D total body photography and convolutional neural networks in a high‐risk population for melanoma under real‐world conditions: A new era of skin cancer screening? Cerminara et al. [32]	Advance imaging for melanoma surveillance	Augmented Intelligence with Deep learning based in imaging 2D CNN FotoFinder automatic total body mapping (ATBM Master) device (FotoFinder System GmbH, Bad Birnbach, Germany, version 3.3.1.0) and 3D CNN Vectra WB360 (Canfield Scientific Inc., Parsippany, New Jersey, United States of America, version 4.7.1) using histology as the ground truth.	Total body photography (TBP) in addition to sequential digital dermoscopy (SSDI) confirmed by dermatologist or histology	Early detection of cutaneous melanoma in high‐risk patients	2D TBP CNN had a sensitivity of 58.3% and a specificity of 97.1%. 3D TBP CNN had a sensitivity of 66.7% and a specificity of 97.7%. When dermatologists predicted melanoma, the 2D‐CNN agreed in 58.33% of cases and the 3D TBP CNN in 66.67% of cases. Both systems had as high specificity as the dermatologists, but were lower than histopathology; and both systems had much lower sensitivity than dermatologists, forcing them to make biopsies to get the diagnosis, but higher specificity.
Implementation of artificial intelligence algorithms for melanoma screening in a primary care setting. Giavina‐Bianchi et al. [33]	AI assisted screening	Deep Learning AI‐assisted melanoma screening in primary care using image‐classification algorithms with DenseNat and EfficientNet.	Clinical high‐quality photography of the lesions analysed followed by dermoscopic images of suspicious areas	Development of an AI system for cutaneous melanoma screening in a primary care setting in two steps: Clinical pathway with photographic images from a teledermatology project for dermatologists, analysed by a DenseNet system and if the result was suspicious a second step of dermoscopy and deep learning analysis with a EfficientNet algorithm.	dermoscopy models accuracy for melanoma was 89.3%, with 0,91 sensitivity and 0,89 specificity clinical model, 84.7% with 0.89 sensitivity and 0.83 specificity. Area under the curve for both models > 0,9.
Using Artificial Intelligence as a Melanoma Screening Tool in Self‐Referred Patients. Crawford et al. [34]	AI assisted screening	Comercial Deep Learning with CNN integrated in the camera FotoFinder Medicam 800HD with dermoscopic images analyzed by MoleAnalyzer Pro Version 6.0	Macroscopic pictures and Dermoscopic images analysis	Development of practical skin cancer screening aid checking accuracy of AI in detecting melanoma in self‐referred patients. The results were endorsed by expert dermatologist comparing sensitivity and specificity, and the suspicious lesions were biopsied and analysed by expert dermatopathologist.	Lack of identification of melanomas in situ which were of difficult diagnose for dermatologists and dermatopathologists. Difficult diagnosis in some body locations. Accuracy; AI: 73.56%/ Dermat: 41.9%–74.72% sensitivity: AI: 64.7%/ Dermat: 47%–82,4% specificity: AI: 75.8%/ Dermat: 31,8%–76,8%.
Validation of a Market‐Approved Artificial Intelligence Mobile Health App for Skin Cancer Screening: A Prospective Multicenter Diagnostic Accuracy Study. Sangers et al. [35]	AI assisted screening	Deep Learning with commercial CNN system for smart phones (Skin Vision)	Convolutional Neural Network (CNN) integrated into a commercial mobile application: CE‐marked mHealth app	Assessment of malignancy risk in skin lesions using smartphone photographs, before visiting a dermatologist.	Overall app sensitivity: 86,9% (82,3 in smooth skin areas‐92,9% in skin fold areas). Notice that sensitivity was higher in iOS devices (91%) than in Android ones (83%), and for benign lesion (80,1%) than for melanoma (45,5%) Overall app specificity: 70,4% (66,2‐74,3% in smooth skin areas) But there were 11,4% of lesions excluded for different reasons, as impossibility to take a good picture with any of the 2 cellular phones available (3,6%) or unsaved photo (3,6%) or missing data (3,38%)
Combining hyperspectral imaging techniques with deep learning to aid in early pathological diagnosis of melanoma C. Tian et al. [36]	Emerging non‐invasive imaging technology for melanoma surveillance using a hyperspectral camera	Deep Learning processed with CNNs Pytorch and Random Forest algorithm compared with the Transfer learning process of the data obtained from the CNNs Pytorch	Hyperspectral imaging (HSI) technology with a GaiaMicro‐G‐V10E‐AZ4 camera obtains spatial image information and spectral information of the sample, and performs spectral analysis on all pixel regions of the space	Evaluation of both procedure of the of hyperspectral imaging analysed by deep learning to discriminate between melanoma and pigmented nevi	Diagnostic accuracy of pigmented tumors and pigmented nevi was 89% for one‐dimensional sample data and 98% for two‐dimensional sample data. The overall performance of the 50‐ layer residual network using transfer learning outperforms the random forest model.
Evaluation of human melanoma and normal formalin paraffin‐fixed samples using Raman and LIBS fused data. Khan, MN et al. [40]	AI assisted screening and classification of melanoma using formalin‐fixed paraffin‐embedded tissue samples analyzed by Raman + LIBS data	Supervised Machine Learning using algorithms ELM, PLS‐DA, and kNN	Spectroscopy‐based classification using fused Raman spectroscopy and laser LIBS supervised classification algorithms.	Classification of melanoma samples versus normal tissue	Raman spectroscopy and LIBS combine can significantly improve the identification and classification accuracy of melanoma and normal specimens when images were analysed by AI. Average accuracy in association of Raman + LIBS: ELM: 99,33%; PLS‐DA: 92% and kNN: 90,33%. Percentage of samples adequately characterized using only Raman spectra: ELM 98%; PLS‐DA: 90%; kNN: 78% Percentage using only LIBS: ELM: 98%; PLS‐DA: 98%; kNN: 93%
A Thermal‐Imaging System and Machine‐Learning Classification Algorithm for Skin Cancer Screening. Mainardi V.et al. [39]	Emerging non‐invasive imaging technology for melanoma surveillance	Supervised Machine Learning with a support vector machine, Random forest and gradient boosting. The system was connected to a thermal‐imaging system with four components: 1. Thermal acquisition system formed by a thermal camera and a standard RGB camera both controlled by a Raspberry Pi 4 Model B+ 2. Thermos‐electronic Cooling system. 3. Control unit and 4. thermal scanner App	Classification algorithm based on thermal features extracted from dynamic thermal images	Differentiation between benign and malignant lesions using thermography and RGB imaging with four supervised classification models: decision tree (DT), gradient boosting (GB), Random forest (RF), and support vector model (SVM) as implemented AI models	Accuracy DT (regression parameters): 70,7%‐79,6% GB (regression parameters + area): 75,9%– 79,6% RF (regression parameters + area): 70,5% –74,1% SVM (exponential parameters): 89,7%–95,5%. Their conclusion is that thermal‐imaging systems could be a non‐invasive low‐cost skin cancer detection system when associated with machine‐learning.

In spite of the classification, the algorithms, the type of determination of information from the skin or biopsies and the procedures were so different in the 8 AI articles that made a rigorous comparative analysis impossible. The only clear conclusion is that the biggest diagnostic accuracy is reached with the SSL systems, which require little effort from the physician but have an intensive, very energy‐consuming training. Probably equivalent in number of hours to that of a senior dermatologist but with an energy and water consumption that makes the system environmentally unsustainable on a community screening scale.

The methods used to obtain the processed images ranged from photographs taken with smart phones, dermoscopy, the combination of photographs and dermoscopy, spectroscopy and thermal imaging. The results of Cerminara et al. [32] allow us to assert that 3D CNN analysis of total body photography associated with histological study of the moles has a superior result to 2D CNN. The superior performance of 3D over 2D CNN approaches for total body photography makes sense, since Breslow depth is one of the main prognostic markers for cutaneous melanoma staging. Hyperspectral imaging applied to histopathological sections also showed promising results, achieving a classification accuracy of 89% with one‐dimensional spectral data using a random forest algorithm and 98% with two‐dimensional spectral‐spatial data using a deep residual network with transfer learning, with the most discriminative spectral differences between melanoma and pigmented nevi located within the 500–675 nm band [36].

Another problem with the AI tools and the systems for data collection used in those articles is that they are very expensive and difficult to find in clinics [32].

Beyond AI, other secondary prevention strategies, including *population screening programs, self‐examination training and public awareness campaigns* showed variable success.

- Screening in a primary care setting or general population screening was associated with earlier diagnosis of melanoma and higher rates of thin lesions [43, 44], suggesting that structured programs can improve diagnostic timing. A lesion‐directed early‐access consultation achieved an overall skin cancer detection rate of 13.2% (4.1% melanoma), rising to 23.6% (9% melanoma) among patients referred by a physician [45]. Additional total‐body examination yielded a low additional detection rate (0.5%) when the index lesion was clinically benign, compared with 8.9% when it was suspicious, supporting a targeted rather than routine total‐body approach in low‐risk patients [45].
- Sargen et al. [48] also achieved successful tumor thickness reduction through longitudinal surveillance in genetically predisposed families; therefore, the findings remain specific to this population group.
- Educational interventions aimed at teaching patients to perform self‐examinations also had some benefits [30, 50, 52, 53], especially in high‐risk populations. Robinson et al. [30] demonstrated that remote self‐examination training produced positive results, but their research included only melanoma survivors and their partners. They constitute motivated patients, which restricts the study's general applicability. The study of Wang et al. [53], emphasizes the importance of the most sceptical populations, whose risk of skin melanoma is exacerbated by very late diagnosis due to cultural reluctance.
- Pharmacy based triage [46] and community campaigns [43, 47, 51, 53], showed potential for increasing accessibility and early referral, but scalability and long‐term efficacy remain uncertain. Further research is needed to tackle these issues.
- Advanced imaging technologies, such as reflectance confocal microscopy and 3D total‐body photography [49], demonstrated high diagnostic accuracy in high‐resource clinical settings, equipped with specialized personnel and infrastructure. These requirements might limit the dissemination and implementation of these instruments.

A new promising method is presented by Huang X et al. [38] with its proposal of Tyrosinase as a local biomarker in skin spots. Tyrosinase detection in blood is an old biomarker [54] associated with malignant melanoma [55] used to detect circulating metastatic melanoma cells [56, 57]. The authors have developed a system combining microneedle patch with nanorattles of gold, silver and platinum, with optimal properties to increase the detection of the Surface‐enhanced Raman spectroscopy method to detect and amplify the colorimetric assay of tyrosinase activity. According to the authors, the path is not producing discomfort or inflammation and adapts easily to all the body surfaces. It could be a promising biomarker if the price of the tool is reasonable.

### 3.3 Quality Assessment

The overall methodological quality of the included studies ranged from moderate to high, varying according to study design and the appraisal tool applied. The 10 observational cohort‐type studies assessed with the Newcastle‐Ottawa Scale (NOS) scored between 6 and 9 out of 9 stars, indicating high quality in nine studies [7, 8, 9] stars [40, 41, 43, 44, 45, 46, 48, 49, 50] and moderate in one study (6 stars) [47], as shown in supplementary Table 7.

The single RCT [30] assessed with the Cochrane RoB2 tool showed an overall low risk of bias across all five domains, as proved in Supplementary Table 8.

The nine observational diagnostic accuracy studies evaluated via QUADAS–2 showed a consistently low risk of bias for the flow‐and‐timing domain. Unclear risk of bias was more frequent for patient selection (4/9 studies) and reference standard (3/9 studies, related to incomplete or non‐independent histological confirmation), and less frequent for the index test domain (2/9 studies). Applicability concerns followed a similar pattern, being most prominent for the patient selection and reference standard domains (Supplementary Table 9).

The cross‐sectional observational studies [42, 51, 52] were assessed with the JBC Critical Appraisal Checklist for Analytical Cross‐Sectional Studies, with two studies rated as high quality (8/8) and one as moderate quality (6/8) as showed in Supplementary Table 10.

Finally, Wang et al. was assessed with the NIH Quality Assessment Tool for Before‐After (Pre‐Post) Studies With No Control Group, receiving an overall rating of Fair, mainly reflecting limited representativeness of the sample and the absence of repeated outcome measurement over time (Supplementary Table 11).

### 3.4 Certainty of Evidence

We applied the GRADE (Grading of Recommendations, Assessment, Development and Evaluation) [29] approach to rate the certainty of evidence across key outcomes. The genetic association with nevi decrement in the aging population is one of the observational‐population based studies with moderate‐low certainty. Aspirin use as a primary preventive strategy for cutaneous melanoma was supported by low‐certainty evidence, reflecting mixed findings and imprecision in effect estimates. Both articles are from the same group of research and need to be confirmed by other assays. AI‐assisted screening tools demonstrated moderate–certainty evidence, showing high sensitivity comparable to dermatologists but variable specificity, raising the potential for over‐referral. Population‐based screening programs and patient self–examination interventions also yielded moderate–certainty evidence, limited mainly by heterogeneity of implementation and indirectness to broader populations. Advanced imaging techniques, such as three–dimensional total body photography and reflectance confocal microscopy, were supported by high certainty evidence in specialist settings, suggesting that they are currently the most reliable tools for the early diagnosis of skin melanoma. The biomarker study also has moderate quality, since the selection of patients is not clear. The detailed GRADE evidence table is provided as Supplementary Table 12.

## 4 Discussion

### 4.1 Principal Findings

This systematic review focus in both primary and secondary prevention strategies for CM. A total of 24 studies were included, comprising 3 studies on primary prevention and 21 on secondary prevention. This difference in the quantity of studies pertaining to each type of prevention could be related to the rise of new technology. Most primary prevention studies date prior to 2020, in fact, our search found a systematic review in primary prevention with sunscreen to prevent skin cancer with dates queried between 1990 and 2017 that found only 4 articles, the last one from 2011 [58]. Clearly, the primordial and primary prevention has produced a change in the tanning fashion with a decrement of the sun exposure and the use of tanning chambers. In spite of the recovery of the ozone layer and the fact that the increment of temperatures makes less pleasant sunbath for long periods, still the risk of sunburn and exposure to high levels of UV radiation remains, particularly among farm or construction workers. It will be of great clinical use the development of a tool to detect the skin tone and the individual mechanisms for protection from UV radiation with increment of melanin and recovery of the damage DNA. It seems that over the last years the focus is set on secondary prevention (screening) due to the rapid technological innovation, such as AI (see also Janda et al. [59] on virtual melanoma checks and Sun et al. [60] on deep–learning–based smartphone applications). But genomics could provide interesting information in a near future. Dessinioti et al. [42] proved that younger people have more nevi that elderly people, being the decrement statistically significant in people older than 51 years (*p* < 0,001), and they also found a bigger decrement in taller people. The idea of association between sets of genes to control an increment of the number of nevi until 35 years old and its regression with aging, was stated in 2006 by Falchi et al. [61] associated with quantitative trait loci on chromosomes 9p2, 5q3‐32 and 2p24. Some studies suggest a possible association between higher nevus count and poorer melanoma survival [62] in male and another study was founding a higher melanoma risk in taller white women [63]. The biggest study about genomics and nevus count found an association between nevus count and a meta‐analysis of genome‐wide association study of almost 37 000 melanoma cases and more than 375 000 controls, identifying 85 loci associated with cutaneous melanoma susceptibility. A total of 31 of them were cutaneous melanoma‐related traits of nevus count and hair color [64]. More studies on genomics are required.

The possible chemopreventive effect of aspirin in hypertensive patients represents a novel finding of potentially great interest in public health, given its accessibility and low cost. However, the evidence is currently of low certainty and must be assessed alongside the known risk of gastrointestinal and cerebral bleeding. Therefore, its use as a primary prevention measure should only be considered after clinical studies that precisely define its risk–benefit balance [29, 40]. There is several evidence of the effect of aspirin on the hallmarks of cancer directly to cancer cells [65], or through the effect on angiogenesis [66], and the action in platelets [67]. The more frequently affected pathway in cutaneous melanoma are (a) MAPK hyperactivation by mutations in *BRAF, NRAS, NF1 or KIT* [68, 69], (b) *PI3K‐AKT* activated in cutaneous melanoma by loss of *PTEN* (c) *CDKN2A*, and (d) *MIFT* pathway. None of them seems affected by aspirin. Other pathway affected in cutaneous melanoma with chances to get benefit from aspirin are *WNT/β* catenin or *NF‐ĸB*, but given the levels of active ingredient required for this effect and the ones normally found in patients taking aspirin or salicylates, we agree with Patrignani et al. (2016) [70] that the most likely was an effect through the platelets and the effect in MMP pathway, platelet inhibition and the consequent activation of the immune system. The effect of aspirin in suppression of cyclooxygenase activity that reduces synthesis of prostaglandin E_2_ has been discovered as the mechanism in colon cancer prevention [71]. But this is still a preliminary hypothesis not allowing a clinical recommendation at all, since in elderly people aspirin even could accelerate cancer progression and data show an increment of mortality [72]. We cannot find an explanation why the protection was detected only in hypertensive patients.

Tyrosinase is the step‐limiting enzyme in the synthesis of melanin by melanocytes, which was stablished last century [73]. The new tool to detect local expression in skin areas that a dermatologist found suspicious, is a rapid way, objective, able to detect cancer in its initial phases if the system is cheap and maintain its sensitivity, specificity and accuracy over time. Could be a more reliable analysis that the current available AI.

With regard to AI applied to screening, the studies included confirm a sensitivity comparable to that of expert dermatologists [30, 37, 47, 49], but with insufficient specificity, which for now limits its widespread implementation. The fact that there are so many different algorithms seems a proof that it is still undergoing development, and probably more research will be necessary to become a reliable tool for diagnoses, in spite of its rapid use.

The educational programmes and the screening performed by expert dermatologists continue to be not only appropriate practices but also necessary for the early diagnosis of patients, thereby increasing the likelihood of a cure and improving quality of life.

### 4.2 Comparison With Previous Research

Previous systematic reviews on cutaneous melanoma prevention have primarily focused on well‐established strategies such as photoprotection, health education, clinical screening, and self‐examination, that also appear successful in this review. Alonso‐Belmonte et al. [74] emphasized the role of traditional measures, such as photoprotection and early detection practices, as the mainstay of melanoma prevention strategies in Europe. Here are also included the potential chemopreventive role of aspirin and the link of genes in CM primary prevention; or the implementation of AI in clinical practice and the tyrosinase determination in suspicious moles as potential tools in the future as new lines of study remarked in our work.

Traditionally, CM prevention has relied on interventions with strong scientific evidence. Among primary prevention strategies, regular sunscreen use reduces significantly melanoma incidence [75], as demonstrated by the RCT by Green et al. [76], and subsequent population studies in Australia confirmed these protective effects at the population level [77, 78]. More recent evaluations have emphasized both the effectiveness and the ongoing challenges of sunscreen promotion, highlighting the need for sustained adherence [76, 79]. Educational campaigns, such as *SunSmart* [77] in Australia, showed the long‐term benefits of structured school‐based interventions, and current evaluations confirm that these programs, when sustained over decades, remain effective in reducing harmful UV exposure among children and adolescents [80, 81].

Secondary prevention is focused on clinical whole‐body examinations, patient self‐examination and dermoscopy, as well as total body photography [82]. Clinical screening in high‐risk groups has been shown to reduce the incidence of thick melanomas [83]. Likewise, self‐examination increases awareness and facilitates earlier consultations, although adherence and diagnostic accuracy remain variable [30, 84]. Dermoscopy, compared with naked‐eye examination, has consistently improved diagnostic accuracy because the images obtained by this system detects changes in the skin structure that are not detectable by visual inspection or pictures [85] and reduced unnecessary excisions, a finding reinforced by recent Cochrane evidence that supports its role as a standard diagnostic tool in dermatology [85, 86]. Probably for that is the technique more often used associated with IA tools. But a deeper standardization of the process seems necessary before considering the system as a real diagnose maker.

Traditional strategies continue to dominate the literature, while AI‐based diagnostic tools are receiving increasing emphasis in secondary prevention. This trend is consistent with recent evaluations of teledermatology (Sangers et al. [35]) and total body photography (Ji‐Xu et al. [87]). However, a recent review on total body photography combined with additional biopsies showed an excessively high number of surgical procedures (Hornung et al. [88]), leading Dr. Kittler to argue that improving melanoma detection requires not only complementary diagnostic techniques but also a well‐defined target population [82].

Against this consolidated background, the results of our review contribute by identifying three new strategies with growing but not established evidence. First, the possible chemopreventive effect of aspirin in hypertensive patients represents a novel pharmacologic approach, though its applicability is currently limited by low‐certainty evidence. Second, AI‐based tools have demonstrated sensitivity comparable to expert dermatologists but remain constrained by limited specificity and scarce validation in diverse populations. Third, skin levels of tyrosinase could be an early biomarker of CM. The relationship between height and protection against CM in older people requires a deep analysis at the genomic level. These findings suggest that while established measures such as sun avoidance, photoprotection and clinical examination should continue to serve as the core of melanoma prevention, emerging strategies like aspirin, if the mechanism of action is demonstrated, tyrosinase determination in skin lesions, genomics discoveries and AI in the hands of expert dermatologists could expand the preventive arsenal in the future, provided that further research confirms their safety, efficacy, and cost‐effectiveness.

## 5 Strengths and Limitations

This review followed PRISMA guidelines. The inclusion of studies from a wide range of geographical regions and encompassing different interventions enhances the generalizability of the findings, and the use of well–established quality assessment tools provided a reliable evaluation of the study quality. Only full original studies with complete methods and results were included; grey literature and incomplete reports were excluded. Several recently published commentaries and perspectives on CM prevention were identified during the search, but these were excluded from the quantitative analysis because they lacked detailed methodology or original data, although they highlight emerging interest in technological solutions and policy interventions [89].

The small number of primary prevention studies limits the strength of conclusions regarding aspirin's protective role [1] as well as the protective genetic background in tall people. Variability in AI–based screening methods and study designs leads to heterogeneity, complicating direct comparisons. Additionally, potential publication bias toward positive results in AI–based diagnostics could have influenced the outcomes. Several studies, particularly those evaluating mobile applications, educational programs, or screening campaigns, did not include control groups or long‐term follow‐up [30, 32, 33, 34, 45], making it difficult to verify the true impact of the interventions. The majority of studies failed to report participants' skin phototypes and did not stratify results accordingly [31, 32, 41, 49], which may limit the generalizability of the results to more diverse populations [10]. Another problem with AI, bigger as the method develops, is the high consumption of energy and water that should be corrected before a massive use of the technique because of health and environmental sustainability. This is particularly relevant for self‐supervised learning systems, which reached the highest diagnostic accuracy among the included studies but rely on computationally intensive training; the energy and computational cost of such training at the scale required for community screening was not addressed by the included studies and would need dedicated evaluation.

Tyrosinase is the limiting step in the synthesis of melanin, which is normally increased in CM; further studies of the wearable biosensor are required to assure its efficiency.

## 6 Implications for Clinical Practice

AI–based tools seem promising in improving early cutaneous melanoma detection due to their high sensitivity, but the low specificity remains a challenge [31, 49]. Still, they are many concerns about their use; but it could be possible that combining AI with clinician oversight could improve accuracy and reduce false positives. The cost and limited availability of some of these AI tools and their associated data‐collection systems in clinical settings is another factor constraining their current implementation. In Population–based screening in high–risk groups also demonstrated benefits, with increased detection of thin melanomas [43] and better patient engagement through self–examination training [33]. Articles shown an improvement in the CNNs, Random forest and other algorithms over the initial machine learning systems, but difficulties with replication persist. It is necessary to ensure the confidentiality of patient data analysed by artificial intelligence, the issue is especially evident in the smartphone images for telediagnoses, and in the use of images of already diagnosed patients to train the different AI systems. Also is required to correct the bias of under‐representation of darker phototypes before proceeding with its clinical use to ensure not discrimination in the accuracy of the system. It is true that the incidence of melanoma in Fitzpatrick phototypes III to VI is lower than in types I and II, but in the countries where AI will start to be available in the future, these phototypes represent a large percentage of people with high sun exposure, which increases their risk of skin melanoma. The absence of images of cutaneous melanoma in darker skin tones will leave those patients, who probably have lower chances of being seen by an expert dermatologist, at a very high risk of being diagnosed at advanced stages of the disease, with the corresponding economic and prognostic implications of a metastatic melanoma. Besides the fact that image quality and perspective, could affect the accuracy of the diagnosis, force to develop rigorous protocols of image acquisition.

Aspirin's potential role as a chemopreventive agent in hypertensive individuals must be checked through different studies before it can be included in clinical guidelines. Detection of Tyrosinase with a wearable biosensor based in nanotechnology seems a promising objective mechanism to differentiate nevus from CM in a very early moment, before the metastasis was established. From an interdisciplinary standpoint, sustained sun‐safety education and policy actions remain essential complements to clinical measures [89].

## 7 Future Research Recommendations

Future research should focus on defining aspirin's chemopreventive role through large–scale randomized trials, with diverse participants and extended follow–up durations. AI diagnostic tools demand increased specificity, with validation across various skin types, lesion subtypes and healthcare settings. The effectiveness of population–based screening approaches needs controlled studies to establish their actual impact on early melanoma detection and mortality reduction. The implementation of standardized protocols through multicentre collaborations would address current methodological heterogeneity to create effective clinical practice guidelines, always as complementary exploration that should be supervised by dermatologists.

## 8 Conclusion

This systematic review assessed the latest evidence on primary and secondary prevention measures for cutaneous melanoma from February 2020 to January 2026.

1. For primary prevention, there is uncertain evidence on aspirin's chemopreventive effect against cutaneous melanoma in uncontrolled hypertensive patients. Further research is needed in this area.
2. An article was detecting a possible link between greater height and faster reduction in the number of nevi in older people. Their hypothesis is a genetic connection that should be explored.
3. The secondary prevention methods using AI–assisted tools with convolutional neural networks applied to total‐body imaging demonstrated high sensitivity in detecting suspicious skin lesions, at a level comparable to expert dermatologists [32, 41, 42]. However, the models displayed a wide range of specificity, which led to concerns about both overdiagnosis and excessive patient referrals [48, 49]. In spite of these limitations, AI tools represent a promising complement to traditional screening always that dermatologists provide supervision [31, 48].
4. Population‐based screening programs, particularly those that integrate self–examination education, have shown increased detection of thin melanomas and improved patient self–examination skills in high–risk groups [33, 43].
5. Future studies should focus on five domains: refining AI models to improve their specificity including a homogeneous number of images of all the Fitzpatrick phototypes; clarifying if aspirin has a role in cutaneous melanoma prevention; finding gene associations to prevent or increase the risk of CM; improving the use of local skin tyrosinase detection of cutaneous melanoma as an early biomarker; and reinforcing public health initiatives to enhance primary prevention strategies, such as self‐examination practices and avoiding exposure to UV radiation.

## Author Contributions

**Maria Palau–del–Valle:** conceptualization, investigation, writing – original draft, methodology, validation, visualization, formal analysis, writing – review and editing. **Elena Bendala–Tufanisco:** conceptualization, investigation, writing – original draft, methodology, validation, visualization, writing – review and editing, formal analysis, project administration. **María Amparo López–Ruiz:** methodology, validation, writing – review and editing, formal analysis, data curation, supervision.

## Funding

The authors have nothing to report.

## Ethics Statement

The authors have nothing to report.

## Conflicts of Interest

The authors declare no conflicts of interest.

## Supporting information


**Supplementary Table 1:** Correlation of the PICO question with the PICO domains.

**Supplementary Table 2:** PICO Criteria of inclusion and exclusion.

**Supplementary Table 3:** Other inclusion and exclusion criteria.

**Supplementary Table 4:** Search strategy for Web of Science database.

**Supplementary Table 5:** Search strategy for PubMed database.

**Supplementary Table 6:** Search strategy for Ebsco Host database.

**Supplementary Table 7:** Quality Assessment for observational studies using the NOS.

**Supplementary Table 8:** Quality Assessment using the RoB2 tool for RCTs.

**Supplementary Table 9:** Quality assessment for Diagnostic Accuracy studies using the QUADAS‐2 scale.

**Supplementary Table 10:** JBI Critical Appraisal Checklist for Analytical Cross‐Sectional Studies.

**Supplementary Table 11:** Quality Assessment using the NIH Quality Assessment Tool for Before‐After (Pre‐Post) Studies With No Control Group for single quality improvement study lacking a control group.

**Supplementary Table 12:** GRADE evidence summary.

## Data Availability

The data supporting this review are available in published journal articles.
