# Supplementary material for: New Primary and Secondary Prevention Measures for Cutaneous Melanoma: A Systematic Review
Source: Cancer Rep (Hoboken). 2026 Oct 1;9(10):e70698. doi: 10.1002/cnr2.70698 (PMC13628457; doi:10.1002/cnr2.70698)
Supplement: Supplementary file 1 [file CNR2-9-e70698-s001.docx]

**Supplementary Table 8.** Quality Assessment using the RoB2 tool for RCTs

| **Study ID** | **D1** | **D2** | **D3** | **D4** | **D5** | **Overall** |
| --- | --- | --- | --- | --- | --- | --- |
| Robinson et al.^39^ | **+** | **+** | **+** | **!** | **+** | **!** |

| **+** | Low risk | D1 | Randomisation process |
| --- | --- | --- | --- |
| **!** | Some concerns | D2 | Deviations from the intended interventions |
| **-** | High risk | D3 | Missing outcome data |
|  |  | D4 | Measurement of the outcome |
|  |  | D5 | Selection of the reported result |
