# Supplementary material for: New Primary and Secondary Prevention Measures for Cutaneous Melanoma: A Systematic Review
Source: Cancer Rep (Hoboken). 2026 Oct 1;9(10):e70698. doi: 10.1002/cnr2.70698 (PMC13628457; doi:10.1002/cnr2.70698)
Supplement: Supplementary file 2 [file CNR2-9-e70698-s002.docx]

**Supplementary Table 5**. Search strategy for PubMed database

| **Database** | **PubMed** |
| --- | --- |
| **Search date** | June 6^th^, 2026 |
| **Search type** | Advanced search |
| **Search terms** | [(Melanoma, Cutaneous Malignant[MeSH Terms]) AND (screening[MeSH Terms]) OR (Melanoma, Cutaneous Malignant[MeSH Terms]) AND (secondary prevention[MeSH Terms]) OR (Melanoma, Cutaneous Malignant[MeSH Terms]) AND (primary prevention[MeSH Terms])](https://pubmed.ncbi.nlm.nih.gov/?term=%28%28%28cutaneous+melanoma%5BMeSH+Terms%5D%29+AND+%28primary+prevention%5BMeSH+Terms%5D%29+AND+%28%28y_5%5BFilter%5D%29+AND+%28ffrft%5BFilter%5D%29+AND+%28english%5BFilter%5D+OR+german%5BFilter%5D+OR+spanish%5BFilter%5D%29%29%29+OR+%28%28cutaneous+melanoma%5BMeSH+Terms%5D%29+AND+%28secondary+prevention%5BMeSH+Terms%5D%29+AND+%28%28y_5%5BFilter%5D%29+AND+%28ffrft%5BFilter%5D%29+AND+%28english%5BFilter%5D+OR+german%5BFilter%5D+OR+spanish%5BFilter%5D%29%29%29%29+OR+%28%28cutaneous+melanoma%5BMeSH+Terms%5D%29+AND+%28screening%5BMeSH+Terms%5D%29+AND+%28%28y_5%5BFilter%5D%29+AND+%28ffrft%5BFilter%5D%29+AND+%28english%5BFilter%5D+OR+german%5BFilter%5D+OR+spanish%5BFilter%5D%29%29%29&filter=datesearch.y_5&filter=simsearch2.ffrft&filter=lang.english&filter=lang.german&filter=lang.spanish&filter=hum_ani.humans&size=100) |
| **Filters** | Since February 2020 to January 2026 English, German, Spanish |
| **Number of results** | 144 |
