# Supplementary material for: New Primary and Secondary Prevention Measures for Cutaneous Melanoma: A Systematic Review
Source: Cancer Rep (Hoboken). 2026 Oct 1;9(10):e70698. doi: 10.1002/cnr2.70698 (PMC13628457; doi:10.1002/cnr2.70698)
Supplement: Supplementary file 3 [file CNR2-9-e70698-s003.docx]

**Supplementary Table 3**. Other inclusion and exclusion criteria.

| **Inclusion** | **Exclusion** |
| --- | --- |
| Cutaneous melanoma | Acral, uveal, mucosal melanoma |
| - Clinical trials  - Randomized controlled trials  - Observational studies | - Systematic reviews  - Meta-analysis  - Grey literature |
| Language: English, Spanish, German |  |
| Works within February 2020 - January 2026 |  |
