# Supplementary material for: New Primary and Secondary Prevention Measures for Cutaneous Melanoma: A Systematic Review
Source: Cancer Rep (Hoboken). 2026 Oct 1;9(10):e70698. doi: 10.1002/cnr2.70698 (PMC13628457; doi:10.1002/cnr2.70698)
Supplement: Supplementary file 4 [file CNR2-9-e70698-s004.docx]

**Supplementary Table 12:** GRADE evidence summary

| Outcome / Intervention | No. S stu.  dies | Study Type | Certainty of Evidence (GRADE) | Justification |
| --- | --- | --- | --- | --- |
| Aspirin (Primary prevention – melanoma incidence) | 2  2 | Observational studies based on a RCT | Low ⬤◯◯◯ | Downgraded for imprecision (wide CI) and inconsistency (mixed results) |
| AI-assisted screening tools (sensitivity & specificity) | 5 | Observational Diagnostic accuracy studies | Moderate ⬤⬤◯◯ | High sensitivity comparable to dermatologists; specificity variable, leading to potential over-referral |
| Population screening | 4 | Observational population-based screening studies | Moderate ⬤⬤◯◯ | Downgraded for inconsistency (programme heterogeneity) |
| Self-exam interventions – detection rate | 3 | Observational + RCT | Moderate ⬤⬤◯◯ | Downgraded for indirectness (motivated survivor populations) |
| Advanced imaging technologies (3D-TBP, RCM, HSI) | 4 | Observational Diagnostic accuracy studies | High ⬤⬤⬤⬤ | Downgraded for indirectness because most studies were conducted in specialist centres and several technologies remain at an early validation stage. |
| Risk stratification and melanoma risk assessment | 5 | Observational  Cross-sectional and observational studies | Moderate ⬤⬤⬤◯ | Consistent associations between phenotypic characteristics and melanoma risk were identified. Downgraded for indirectness because evidence was observational and no intervention was evaluated. |
| Biomarker determination with nanotechnology | 1 | Observational Diagnostic accuracy study | Moderate ⬤⬤⬤◯ | Objective determination of the biomarker with spectroscopic well-developed technique in a wearable biosensor introducing nanotechnology. Not clear patient selection and uncertainty of tyrosine expression in very malignant melanoma with a high degree of cellular undifferentiation. |
