# Supplementary material for: New Primary and Secondary Prevention Measures for Cutaneous Melanoma: A Systematic Review
Source: Cancer Rep (Hoboken). 2026 Oct 1;9(10):e70698. doi: 10.1002/cnr2.70698 (PMC13628457; doi:10.1002/cnr2.70698)
Supplement: Supplementary file 5 [file CNR2-9-e70698-s005.docx]

**Supplementary Table 9**. Quality assessment for Diagnostic Accuracy studies using the QUADAS-2 scale

| **Study** | **RISK OF BIAS** | | | | **APPLICABILITY CONCERNS** | | |
| --- | --- | --- | --- | --- | --- | --- | --- |
|  | **Patient selection** | **Index test** | **Reference standard** | **Flow and timing** | **Patient selection** | **Index test** | **Reference standard** |
| **Useini et al.^33^** | ☺ | ☺ | ☹ | ? | ☺ | ☺ | ☹ |
| **Cerminara et al.^35^** | ☺ | ? | ☺ | ☺ | ☺ | ☺ | ☺ |
| **Giavina-Bianchi et al.^37^** | ? | ☺ | ? | ☺ | ? | ☺ | ? |
| **Crawford et al.^44^** | ☺ | ☺ | ? | ☺ | ☺ | ☺ | ☺ |
| **Sangers et al.^45^** | ☺ | ☺ | ? | ☺ | ☺ | ☺ | ? |
| **Tian et al.^47^** | ? | ☺ | ☺ | ☺ | ? | ☺ | ☺ |
| **Mainardi et al.^53^** | ? | ☺ | ☺ | ☺ | ? | ☺ | ☺ |
| **Khan et al.^50^** | ☹ | ? | ☺ | ☺ | ☹ | ? | ☺ |
| **Huang et al.^52^** | ? | ☺ | ☺ | ☺ | ? | ☺ | ☺ |

☺Low Risk	☹High Risk	? Unclear Risk
