# Supplementary material for: New Primary and Secondary Prevention Measures for Cutaneous Melanoma: A Systematic Review
Source: Cancer Rep (Hoboken). 2026 Oct 1;9(10):e70698. doi: 10.1002/cnr2.70698 (PMC13628457; doi:10.1002/cnr2.70698)
Supplement: Supplementary file 6 [file CNR2-9-e70698-s006.docx]

**Supplementary Table 4.** Search strategy for Web of Science database

| **Database** | **Web of Science** |
| --- | --- |
| **Search date** | June 6th, 2026 |
| **Search type** | Advanced search |
| **Search terms** | [TS=(("cutaneous melanoma" AND "primary prevention") OR ("cutaneous melanoma" AND "secondary prevention") OR ("cutaneous melanoma" AND "screening"))](https://www.webofscience.com/wos/alldb/summary/aa60ceb6-956b-4c70-9f2d-26c7931a7cdf-0146ff4408/relevance/1) |
| **Filters** | NOT Document Types: Review Article  Languages: English or German  Since February 2020 to January 2026 |
| **Number of results** | 114 |
