# Supplementary material for: New Primary and Secondary Prevention Measures for Cutaneous Melanoma: A Systematic Review
Source: Cancer Rep (Hoboken). 2026 Oct 1;9(10):e70698. doi: 10.1002/cnr2.70698 (PMC13628457; doi:10.1002/cnr2.70698)
Supplement: Supplementary file 7 [file CNR2-9-e70698-s007.docx]

**Supplementary Table 2**. PICO Criteria of inclusion and exclusion.

|  | **Inclusion** | **Exclusion** |
| --- | --- | --- |
| ***Population*** | - General population - Any age - No melanoma diagnosed | - Diagnosed melanoma - Not human tested |
| ***Intervention*** | - Primary and secondary prevention measures | - Active treatment - Tertiary prevention/recurrence |
| ***Comparison*** | - Basal observation - Another comparator |  |
| ***Outcome*** | - Reduction in CM incidence following a primary prevention intervention - Screening efficiency - Thinner melanomas detected at diagnosis | - No lesser CM incidence or thinner at diagnosis. - No better screening effectiveness |
