# Supplementary material for: New Primary and Secondary Prevention Measures for Cutaneous Melanoma: A Systematic Review
Source: Cancer Rep (Hoboken). 2026 Oct 1;9(10):e70698. doi: 10.1002/cnr2.70698 (PMC13628457; doi:10.1002/cnr2.70698)
Supplement: Supplementary file 8 [file CNR2-9-e70698-s008.docx]

**Supplementary Table 1.** Correlation of the PICO question with the PICO domains.

| **Population** | **Intervention** | **Comparison** | **Outcome** |
| --- | --- | --- | --- |
| General population | Primary and secondary prevention measures | Basal observation or another 1ry or 2ry intervention | Reduction in CM incidence or thickness at diagnosis |
