# Supplementary material for: New Primary and Secondary Prevention Measures for Cutaneous Melanoma: A Systematic Review
Source: Cancer Rep (Hoboken). 2026 Oct 1;9(10):e70698. doi: 10.1002/cnr2.70698 (PMC13628457; doi:10.1002/cnr2.70698)
Supplement: Supplementary file 9 [file CNR2-9-e70698-s009.docx]

**Supplementary Table 7.** Quality Assessment for observational studies using the NOS

|  |  | M.K. Yan et al. ^30^ (Association between…) | | M.K. Yan et al.^31^ (Effect of aspirin…) | Lee et al.^34^ | M. Matsumoto et al. ^36^ | Mylle et al.^38^ | A. González et al.^40^ | L. Demidov et al.^41^ | Sargen et al.^42^ | Hobelsberger et al.^43^ | Duarte et al.^46^ |
| --- | --- | --- | --- | --- | --- | --- | --- | --- | --- | --- | --- | --- |
| **Selection** | Representativeness of the exposed cohort | | ★ | ★ | ★ | ★ | ★ | ★ | ★ | ★ | ★ | ★ |
|  | Selection of the non-exposed cohort | | ★ | ★ | X | ★ | ★ | X | X | ★ | ★ | ★ |
|  | Ascertainment of exposure | | ★ | ★ | ★ | ★ | ★ | ★ | ★ | ★ | ★ | ★ |
|  | Demonstration that outcome was not present at start | | ★ | ★ | ★ | ★ | ★ | ★ | ★ | ★ | ★ | ★ |
| **Comparability** | Comparability of cohorts based on design or analysis | | ★ | ★ | ★★ | ★★ | ★★ | ★★ | ★★ | ★★ | ★★ | ★★ |
| **Outcome** | Assessment of outcome | | ★ | ★ | ★ | ★ | ★ | ★ | ★ | ★ | ★ | ★ |
|  | Follow-up was long enough for outcomes to occur | | ★ | ★ | ★ | ★ | ★ | ★ | X | ★ | ★ | X |
|  | Adequacy of follow-up of cohorts | | ★ | ★ | ★ | ★ | ★ | ★ | X | ★ | ★ | X |
| **Total score** | | | **8** | **8** | **8** | **9** | **9** | **8** | **6** | **9** | **9** | **7** |

*One star can be given for each criteria met, except for comparability, where a maximum of 2 stars can be given. The total score is measured out of 9 stars (****7–9 stars*** *→ high quality,* ***5–6 stars*** *→ moderate quality,* ***≤4 stars*** *→ low quality)*
