# Supplementary material for: New Primary and Secondary Prevention Measures for Cutaneous Melanoma: A Systematic Review
Source: Cancer Rep (Hoboken). 2026 Oct 1;9(10):e70698. doi: 10.1002/cnr2.70698 (PMC13628457; doi:10.1002/cnr2.70698)
Supplement: Supplementary file 10 [file CNR2-9-e70698-s010.docx]

# ***Supplementary Table 11:*** *Quality Assessment using the NIH Quality Assessment Tool for Before-After (Pre-Post) Studies With No Control Group for single quality improvement study lacking a control group.*

|  |  | **Wang et al.** |
| --- | --- | --- |
| **#** | **Criteria** | **Rating** |
| 1 | Was the study question or objective clearly stated? | Yes |
| 2 | Were eligibility/selection criteria for the study population pre-specified and clearly described? | Yes |
| 3 | Were the participants in the study representative of those who would be eligible for the intervention in the general population of interest? | No |
| 4 | Were all eligible participants who met the pre-specified entry criteria enrolled? | Cannot Determine |
| 5 | Was the sample size sufficient to provide confidence in the findings? | Yes |
| 6 | Was the intervention clearly described and delivered consistently across the study population? | Yes |
| 7 | Were the outcome measures pre-specified, clearly defined, valid, reliable, and assessed consistently across all participants? | Yes |
| 8 | Were the people assessing the outcomes blinded to the participants' exposures/interventions? | Not applicable |
| 9 | Was the loss to follow-up after baseline 20% or less? | Yes |
| 10 | Were statistical methods appropriate to assess the pre-post change in the primary outcome? | Yes |
| 11 | Was the outcome assessed multiple times before and after the intervention (trend assessment)? | No |
| 12 | If the intervention was delivered at a group level, was there an adequate analysis accounting for the effect of the intervention on individuals (clustering)? | No |
| **Overall appraisal** | | **Fair** |

*There is not a rigid numerical cutoff formula provided for this assessment. Reviewers compile the responses to assign an overall subjective quality rating between: Good, Fair and Poor.*
