# Supplementary material for: New Primary and Secondary Prevention Measures for Cutaneous Melanoma: A Systematic Review
Source: Cancer Rep (Hoboken). 2026 Oct 1;9(10):e70698. doi: 10.1002/cnr2.70698 (PMC13628457; doi:10.1002/cnr2.70698)
Supplement: Supplementary file 11 [file CNR2-9-e70698-s011.docx]

**Suplemmentary Table 10:** JBI Critical Appraisal Checklist for Analytical Cross-Sectional Studies

|  | **Beaulieu et al.^48^** | **Mannino et al.^49^** | **Dessinioti et al.^32^** |
| --- | --- | --- | --- |
| **1. Were the criteria for inclusion in the sample clearly defined?** | Yes | Yes | Yes |
| **2. Were the study subjects and the setting described in detail?** | Yes | Yes | Yes |
| **3. Was the exposure measured in a valid and reliable way?** | Yes | Yes | Yes |
| **4. Were objective, standard criteria used for measurement of the condition?** | Unclear | Yes | Yes |
| **5. Were confounding factors identified?** | Yes | Yes | Yes |
| **6. Were strategies to deal with confounding factors stated?** | Yes | Yes | Yes |
| **7. Were the outcomes measured in a valid and reliable way?** | Unclear | Yes | Yes |
| **8. Was appropriate statistical analysis used?** | Yes | Yes | Yes |
| **Overall appraisal** | **6/8** | **8/8** | **8/8** |

*One point was assigned for each criterion met. The total score was measured out of 8 points (****7–8 points*** *→ high methodological quality,* ***5–6 points*** *→ moderate methodological quality****, ≤4 points*** *→ low methodological quality).*
