# Supplementary material for: New Primary and Secondary Prevention Measures for Cutaneous Melanoma: A Systematic Review
Source: Cancer Rep (Hoboken). 2026 Oct 1;9(10):e70698. doi: 10.1002/cnr2.70698 (PMC13628457; doi:10.1002/cnr2.70698)
Supplement: Supplementary file 12 [file CNR2-9-e70698-s012.docx]

**Supplementary Table 6**. Search strategy for Ebsco Host database

| **Database** | **Ebsco Host** |
| --- | --- |
| **Search date** | June 6th, 2026 |
| **Search type** | Advanced search |
| **Search terms** | [("cutaneous melanoma" AND ("primary prevention" or "early intervention" or "preventive health services")) OR ("cutaneous melanoma" AND ("secondary prevention" or "screening"))](https://research.ebsco.com/c/y43uig/search/results?db=a9h%2Cpsyh%2Cbvh%2Cbth%2Crzh%2Ccms%2Cddh%2Cnlebk%2Ceoah%2Ceric%2Cfua%2Chlh%2Ccmedm%2Ckah%2Cmzh%2Cddu%2Clsdpd%2Cpbh%2Cbwh%2Cs3h%2Cser&expanders=concept&limiters=FT%3AY%2CDT1%3A2020-02-03%2F2025-02-03&q=%28%22cutaneous%20melanoma%22%20AND%20%28%22primary%20prevention%22%20or%20%22early%20intervention%22%20or%20%22preventive%20health%20services%22%29%29%20OR%20%28%22cutaneous%20melanoma%22%20AND%20%28%22secondary%20prevention%22%20or%20%22screening%22%29%29&searchMode=boolean&sort=relevance) |
| **Filters** | Since February 2020 to January 2026 |
| **Number of results** | 101 |
